# Global Genomic Analysis Reveals Spatial Patterns and Temporal Trends of Priority-Antimicrobial Resistance in *Salmonella* *enterica* Isolates from Aquatic Animals

**DOI:** 10.3390/vetsci13090868

**Published:** 2026-08-26

**Authors:** Ziru Yang, Shuqi Yan, Limin Zhang, Chaochao Zhao, Jiangtao Wang, Jiale Chen, Yue Liu, Xuebin Xu, Yanan Wang

**Affiliations:** 1 International Joint Research Center of National Animal Immunology, College of Veterinary Medicine, Henan Agricultural University, Zhengzhou 450046, China; 2 Longhu Laboratory of Advanced Immunology, Zhengzhou 450046, China; 3 Division of Pathogen Testing and Analysis, Shanghai Municipal Center for Disease Control and Prevention, Shanghai 201107, China; 4 Beijing Key Laboratory of Antimicrobial-Resistant Pathogen Microbiology and AI-Empowered Containment, Institute of Microbiology, Chinese Academy of Sciences (CAS), Beijing 100101, China

**Keywords:** aquaculture, *Salmonella enterica*, antimicrobial resistance, genomic surveillance, One Health

## Abstract

Antimicrobial resistance (AMR) in *Salmonella enterica* from aquatic animals represents an emerging food safety and public health concern, yet the global distribution patterns and trends remain insufficiently understood. Here, we performed a global genomic survey of *Salmonella* from aquatic animals using 2911 genomes collected from 67 countries across six continents between 1974 and 2025. These genomes represented 194 serovars and 364 sequence types. Notably, the dataset exhibited unbalanced geographical sampling, with Asian isolates accounting for 69.98% of all strains, which may limit the generalizability of our global trends. Globally, resistance to WHO-recommended clinically important antimicrobials showed substantial variation among antibiotic classes, with resistance rates for azithromycin (2.10%), cefepime (2.89%), cefotaxime (2.71%), ceftazidime (2.71%), and ceftriaxone (0.62%). Geographical differences were observed, with isolates from Asia exhibiting higher resistance levels, particularly for ciprofloxacin (13.50%) and doxycycline (18.02%). AMR temporal trends differed across geographical regions. A total of 111 acquired ARGs and 10 resistance-associated chromosomal mutations were identified, accompanied by changes in the distribution of resistance determinants. Collectively, this study provides a global genomic perspective on priority AMR in *Salmonella* from aquatic animals and highlights the importance of integrating aquaculture, environmental, and public health surveillance to monitor and mitigate AMR risks within the One Health framework.

## Simple Summary

*Salmonella* is a widespread foodborne pathogenic bacterium across the globe. It poses serious threats to food safety, aquaculture, and public health. To explore the temporal trends and regional heterogeneity of World Health Organization-recommended priority antibiotic resistance in *Salmonella* from aquatic animals, this study analyzed the genomic data of 2911 *Salmonella* strains isolated from aquatic animals worldwide. The study found that antibiotic resistance patterns changed over time, with increased resistance to several priority drugs, although these trends differed across geographic regions. Overall, these findings can provide an important baseline for rational medication and the prevention of antibiotic resistance risks in aquaculture.

## 1. Introduction

*Salmonella enterica* (*S. enterica*), a Gram-negative pathogen within the family Enterobacteriaceae, is a major cause of foodborne infections worldwide [1]. The Global Burden of Disease (GBD) Study estimates that *S. enterica* is responsible for approximately 420,300 annual deaths worldwide [2]. Children under 5 years old, elderly individuals, and immunocompromised individuals are high-risk groups [3]. In China, *Salmonella* has consistently ranked among the top foodborne pathogens, making it one of the major causes of foodborne disease outbreaks and imposing a considerable burden on clinical treatment, disease control, and socioeconomic development [4].

The rapid emergence and spread of multidrug-resistant (MDR) *Salmonella* have become a major public health concern worldwide [5]. Fluoroquinolones, third-generation cephalosporins (3GCs), such as cefotaxime and ceftriaxone, and azithromycin are among the recommended therapeutic options for the treatment of moderate to severe salmonellosis; however, the increasing emergence of resistance to these clinically important antimicrobials has posed substantial challenges to effective treatment and narrowed available therapeutic choices [6,7,8,9]. *S*. *enterica* has been categorized as a priority pathogen in the 2024 World Health Organization Bacterial Priority Pathogens List (WHO BPPL) [6]. Patients infected with resistant *Salmonella* often require longer hospitalization and incur higher medical costs, and also face higher risks of severe invasive illnesses such as bacteremia, meningitis, and osteomyelitis, leading to increased mortality [10,11]. In addition, antimicrobial resistance (AMR) can disseminate rapidly among bacterial populations through horizontal gene transfer mediated by mobile genetic elements (MGEs), such as plasmids, insertion sequences, and transposons, which facilitate the acquisition, mobilization, and persistence of antibiotic resistance genes (ARGs) [12,13]. Antimicrobial-resistant *Salmonella* can be transmitted between animals and humans through the food chain, posing continuous challenges to clinical treatment and public health [5,14].

Global aquaculture has expanded rapidly over the past few decades and has become one of the fastest-growing animal food production sectors [15]. In 2022, global farmed aquatic animal production reached 94.4 million tonnes, surpassing wild-caught aquatic production for the first time [15]. China has remained the world’s largest aquaculture producer for more than three decades, accounting for approximately 55% of global aquaculture production [15]. To prevent and treat bacterial diseases under intensive farming conditions, antimicrobials are commonly used in aquaculture. Such practices have been recognized as potential contributors to the emergence, selection, and dissemination of AMR in aquatic environments [16]. Several clinically important antimicrobial classes, including fluoroquinolones, macrolides, and β-lactams, are also used in aquaculture, where antimicrobial exposure may exert selective pressure and contribute to the emergence and dissemination of AMR bacteria and resistance genes [16,17,18,19].

*Salmonella* can survive in aquaculture water, sediments, feed, and fry and spread among different aquatic species, including fish, shrimp, crabs, mollusks, and turtles [20]. Aquatic-derived *Salmonella* can be transmitted to humans through contaminated aquatic products, water exposure, and international trade. This public health concern is amplified by the fact that aquaculture environments have been well established as significant reservoirs for AMR *Salmonella* [21]. Aquatic animals and aquaculture environments may serve as important reservoirs for AMR bacteria, including *Salmonella*, posing potential public health risks under the One Health framework [16,22,23]. In particular, resistance to clinically important antibiotics directly affects treatment outcomes in patients with severe salmonellosis [24].

Although transferable resistance determinants and AMR dissemination in *Salmonella* have been widely reported [25,26], the distribution and evolutionary dynamics of clinically important resistance in aquatic-derived *Salmonella* remain insufficiently understood. Regional differences in antimicrobial usage, farming modes, and surveillance systems lead to geographically heterogeneous AMR patterns [27,28]. These factors highlight the need for a comprehensive understanding of the distribution, evolution, and transmission potential of AMR in aquatic-derived *Salmonella*.

Previous studies have investigated AMR in livestock- and poultry-derived *Salmonella*, revealing increasing resistance rates, widespread MDR, and the dissemination of plasmid-mediated resistance genes [5,29]. Recent global genomic studies have provided important insights into the spatiotemporal evolution and drivers of AMR in *Salmonella*; however, aquatic-associated lineages and their contribution to clinically relevant resistance remain insufficiently characterized [5,28]. In addition, several studies have characterized AMR profiles and ARGs in aquatic isolates [5,20,30], yet no previous study has exclusively assessed the global spatiotemporal distribution of resistance to WHO priority antimicrobials in aquatic animal-derived *Salmonella* strains, while also considering geographic regions, serovars, and STs. In this study, we analyzed global genomics (*n* = 2911) to clarify the distribution, evolution, and dissemination of clinically important AMR in aquatic-derived *Salmonella*.

## 2. Materials and Methods

### 2.1. Data Collection of Salmonella Genomes

*S. enterica* genomes were retrieved from the NCBI GenBank Genome Database (https://www.ncbi.nlm.nih.gov/datasets/genome/salmonella_enterica) on 14 February 2025, and integrated with genomic sequences from the Chinese Local *Salmonella* Genome Database (https://nmdc.cn/clsgdbv2, accessed on 14 February 2025) [7,31]. Relevant sample information was extracted from the NCBI BioSample database (https://www.ncbi.nlm.nih.gov/biosample/, accessed on 14 February 2025). After metadata screening and systematic genome quality control based on previously described criteria [5,32], a total of 2911 aquatic animal-derived *S. enterica* isolates were enrolled for AMR analysis. Genomes with complete metadata regarding isolation source, sampling location, and collection date were retained for subsequent analyses. Sequence types (STs) and serovars were determined using three bioinformatic tools, including Multilocus Sequence Typing (MLST v2.22.0, https://github.com/tseemann/mlst, accessed on 14 February 2025) [33], *Salmonella* In Silico Typing Resource (SISTR v1.1.2, https://github.com/phac-nml/sistr_cmd, accessed on 14 February 2025) [34], and the whole-genome serotyping pipeline SeqSero2 (v1.3.1, https://github.com/denglab/SeqSero2, accessed on 14 February 2025) [35]. Complete metadata and accession numbers are presented in Supplementary Table S1. The prevalence and geographic distribution of these isolates were characterized across different sampling periods, continents, countries, host species, STs, and serovars.

### 2.2. Bioinformatic Analysis

The newly generated genomes in this study were sequenced using the Illumina NovaSeq 6000 platform, and detailed sequencing protocols were described in our previous study [31]. Additional publicly available *Salmonella* genomic assemblies were retrieved from the NCBI database for comparative genomic analysis. Fastp v0.23.4 was applied for raw data quality trimming under the parameters “-W 4 -M 20 -n 5 -c -l 50 -w 3” (https://github.com/OpenGene/fastp, accessed on 14 February 2025) [36]. QUAST v5.3.0 was utilized to evaluate the overall quality of genome assemblies (https://github.com/ablab/quast, accessed on 14 February 2025) [37]. Genome assemblies with a total length of 4–6 Mb were retained for subsequent analyses, and all assembly statistics are summarized in Supplementary Table S2. Clean sequencing reads were assembled using Unicycler v0.4.8 (https://github.com/rrwick/Unicycler, accessed on 14 February 2025) [38]. Acquired AMR determinants and chromosomal gene mutations mediating AMR within genomic sequences were screened via ResFinder v4.1 (https://bitbucket.org/genomicepidemiology/resfinder/src/master/, accessed on 14 February 2025) integrated with the ResFinder database and PointFinder module [39]. All *S*. *enterica* isolates with complete serovar identification were retained for downstream AMR analyses. Isolates lacking detectable acquired resistance genes or resistance-associated chromosomal mutations were not excluded and were considered negative for the corresponding AMR determinants. The ResFinder analysis was performed with a minimum sequence identity of 95% and a minimum coverage of 90%, as recommended in previous studies [5,7,25,32]. Chromosomal resistance-associated mutations were identified using the PointFinder module with the *Salmonella enterica*-specific database [40]. Only mutations annotated as resistance-associated in the PointFinder database were retained for downstream analysis. The AMR profiles of each isolate were predicted based on the correspondence between detected resistance genes and antimicrobial classes according to the ResFinder database, PointFinder annotations, and previously reported genotype–phenotype associations [39,40,41]. When multiple resistance determinants associated with the same antimicrobial class were detected, the presence of at least one relevant resistance determinant was considered sufficient to predict resistance to the corresponding antimicrobial class.

### 2.3. Statistical Analysis and Data Visualization

Strains were divided into six distinct temporal groups: 1974–2000, 2001–2005, 2006–2010, 2011–2015, 2016–2020, and 2021–2025. A longer time window spanning 1974–2000 was applied owing to the limited availability of public *Salmonella* genomic data generated before 2001. We adopted successive five-year periods for later collections to standardise temporal resolution and guarantee enough isolates per group for credible cross-period comparisons of AMR prevalence. The 95% Wilson score confidence intervals for phenotypic AMR prevalence of *Salmonella* in each subgroup were calculated. All prevalence estimates and corresponding confidence intervals are presented in Supplementary Table S3. For temporaltrend analysis, only time intervals containing ≥5 isolates were included for trend modelling. Formal pairwise statistical testing across time groups was not conducted. Temporal trends in AMR prevalence were assessed visually using prevalence estimates and their 95% Wilson score confidence intervals; hence, no multiple testing correction was applied. The global distribution map of *Salmonella* was generated based on the standard map service system of the Ministry of Natural Resources of China (http://bzdt.ch.mnr.gov.cn, accessed on 14 February 2025). Map review No. GS(2021)5449, with no modification to national boundary features. The prevalence of AMR was calculated as the proportion of resistant isolates and visualized using GraphPad Prism v9.0. Heat maps were generated on the ImageGP platform (http://www.ehbio.com/ImageGP/, accessed on 14 February 2025) [42].

## 3. Results

### 3.1. Overview of Global Salmonella Genomes from Aquatic Animals

**Figure 1:**
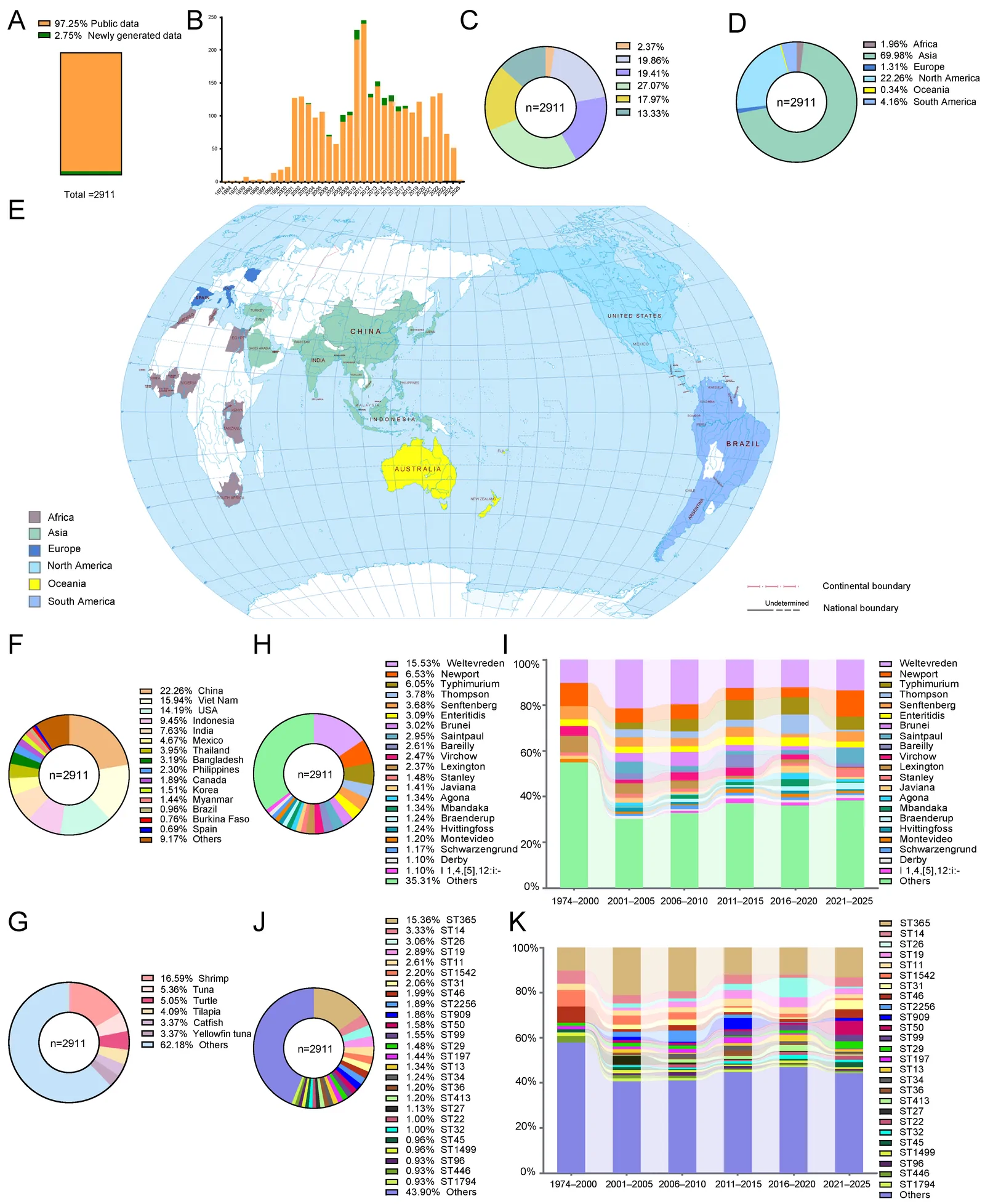
Geographical, temporal, source, serovar, and sequence type distribution of global *Salmonella* genomes from aquatic animals. (**A**) Proportion of *Salmonella* genomes obtained from public databases and newly sequenced in this study. (**B**) Annual dynamics of *Salmonella* genomes from public databases and newly sequenced isolates in this study. (**C**) Temporal distribution of *Salmonella* genomes across different periods. (**D**) Continental distribution of *Salmonella* genomes across six continents. (**E**) Geographical distribution of 2911 *Salmonella* genomes collected from 67 countries and regions across six continents. Base map is derived from the standard map of the Ministry of Natural Resources of China (Map review No. GS (2021) 5449), with no modification to national boundary features. (**F**) Distribution of *Salmonella* isolates in the top 16 countries. (**G**) Genomic profiles from different sources. (**H**) The top 21 dominant serovars in the global *Salmonella* genome dataset. (**I**) Temporal trends of the top 21 dominant serovars across different periods. (**J**) The top 26 dominant STs in the global *Salmonella* genome dataset. (**K**) Temporal trends of the top 26 STs across different periods.

A total of 2911 *Salmonella* genomes originating from aquatic animals were assembled into the present dataset. Eighty isolates (2.75%) were recovered from four provincial-level regions across China, while the remaining 2831 high-quality genomic sequences (97.25%) were retrieved from the NCBI database (Figure 1A). Newly generated whole-genome sequencing data were mainly collected between 2008 and 2017, whereas publicly available genome data covered a broader time frame from 2001 to 2022 (Figure 1B). Overall, the full collection spanned isolation years ranging from 1974 to 2025 (Figure 1C). In chronological grouping, 69 isolates dated back to 1974–2000. The total reached 578 in 2001–2005, representing a more than seven-fold increase, and 788 isolates were obtained during 2006–2015 (Figure 1C).

Geographic distribution analysis revealed that Asian isolates accounted for 69.98% of all isolates, ranking first worldwide. North America constituted the second-largest group at 22.26%, followed by South America (4.16%). Isolates from Europe, Africa, and Oceania occupied relatively small fractions, with proportions of 1.31%, 1.96%, and 0.34%, respectively (Figure 1D). This geographic imbalance indicates that our AMR distribution results predominantly reflect the epidemiological features of Asian *Salmonella*, and we caution against excessive generalization of these findings to other continents. These isolates were sampled from 67 countries across six continents. Specifically, the number of sampled countries was 20 in Asia, 14 in Africa, 13 in North America, 10 in South America, 7 in Europe, and 3 in Oceania. Sampling coverage at the national level was highest in North America and South America, followed by Asia, while the other three continents showed lower coverage (Figure 1E). China contributed the largest number of isolates (n = 648), followed by Viet Nam (n = 464), the USA (n = 413), Indonesia (n = 275), and India (n = 222) (Figure 1F). Host source statistics indicated that shrimp was the predominant host, accounting for 16.59% of all isolates. Other prevalent aquatic animal hosts included tuna, turtles, tilapia, catfish, and yellowfin tuna, with relative abundances of 5.36%, 5.05%, 4.09%, 3.37%, and 3.37%, respectively (Figure 1G).

Serovar analysis identified 194 distinct serovars among these aquatic animal-derived isolates. *S*. Weltevreden emerged as the dominant serovar, with 452 isolates (15.53% of all isolates). *S*. Newport (n = 190) and *S*. Typhimurium (n = 176) were the second and third most dominant serovars in this collection (Figure 1H). The prevalence of *S*. Weltevreden has increased consistently since 2011. This serovar persisted across the entire study timeline and maintained a high prevalence in different periods, with the highest prevalence observed during 2001–2010. *S*. Newport exhibited high prevalence before 2000, followed by a subsequent decline, but rebounded in 2021–2025, indicating its long-term endemic circulation. Meanwhile, the prevalence of *S*. Typhimurium increased steadily from 1974 to 2020 (Figure 1I). MLST assigned these isolates to 364 distinct STs. ST365 was the most prevalent genotype, represented by 447 isolates. Other major STs included ST14 (n = 97), ST26 (n = 89), ST19 (n = 84) and ST11 (n = 76) (Figure 1J). ST14, ST26, and other STs exhibited a gradual increase in prevalence after 2010. Collectively, the dominant STs of aquatic animal-derived *Salmonella* underwent obvious succession across years (Figure 1K).

### 3.2. Regional Heterogeneity in Clinically Priority Antibiotic-Resistant Salmonella Across Regions and Dominant Clones

Overall, AMR varied considerably among aquatic *Salmonella* isolates across different continents (Table 1). Globally, the resistance rates to azithromycin, cefepime, cefotaxime, ceftazidime, and ceftriaxone were 2.10%, 2.89%, 2.71%, 2.71%, and 0.62%, respectively (Table 1). Resistance to colistin and fosfomycin was 0.48% and 4.29%, respectively. *Salmonella* isolates from Asia exhibited the highest overall resistance levels, with resistance rates of 13.50% to ciprofloxacin and 18.02% to doxycycline. Isolates from Europe showed the highest resistance to fosfomycin (18.42%) and azithromycin (5.26%) and also exhibited relatively high resistance to doxycycline (10.53%). In South America, resistance to ciprofloxacin and doxycycline was 9.92% and 5.79%, respectively. In contrast, isolates from Africa generally showed low resistance rates, with only tetracycline (7.02%) and fosfomycin (5.26%) exhibiting moderate resistance. Isolates from North America and Oceania displayed the lowest overall resistance levels. In North America, the resistance rate to doxycycline was 6.33%, whereas isolates from Oceania showed a fosfomycin resistance rate of 10.00% (Table 1). AMR also varied markedly among aquatic *Salmonella* isolates from different countries. In China, resistance to doxycycline reached 41.36%, while resistance to ciprofloxacin exceeded 30.00%. Isolates from Korea exhibited a high resistance rate to fosfomycin (27.27%).

Distinct variations in AMR prevalence were also observed among different serovars and STs. The highest azithromycin resistance was found in *S*. Thompson (22.73%) and ST26 (28.09%). For cefotaxime and ceftazidime, resistance rates reached 32.73% in *S*. Thompson and 40.45% in ST26. Cefepime resistance was mainly detected in ST19 (17.86%) and *S*. Typhimurium (16.48%). Ciprofloxacin resistance was most prevalent in ST26 (38.20%), followed by *S*. Thompson (30.91%), *S*. Typhimurium (28.41%), and ST19 (25.00%). Doxycycline resistance showed relatively high levels among several lineages, with the highest rates observed in ST26 (59.55%), *S*. Thompson (48.18%), ST19 (40.48%), and *S*. Typhimurium (30.68%). Ceftriaxone resistance was identified in *S*. Typhimurium, *S*. Enteritidis, and ST11. Colistin resistance was detected only in *S*. Typhimurium (1.14%) (Table 2).

### 3.3. Dynamic Changes in the Resistance Prevalence of Salmonella Against Clinically Priority Antibiotics

**Figure 2:**
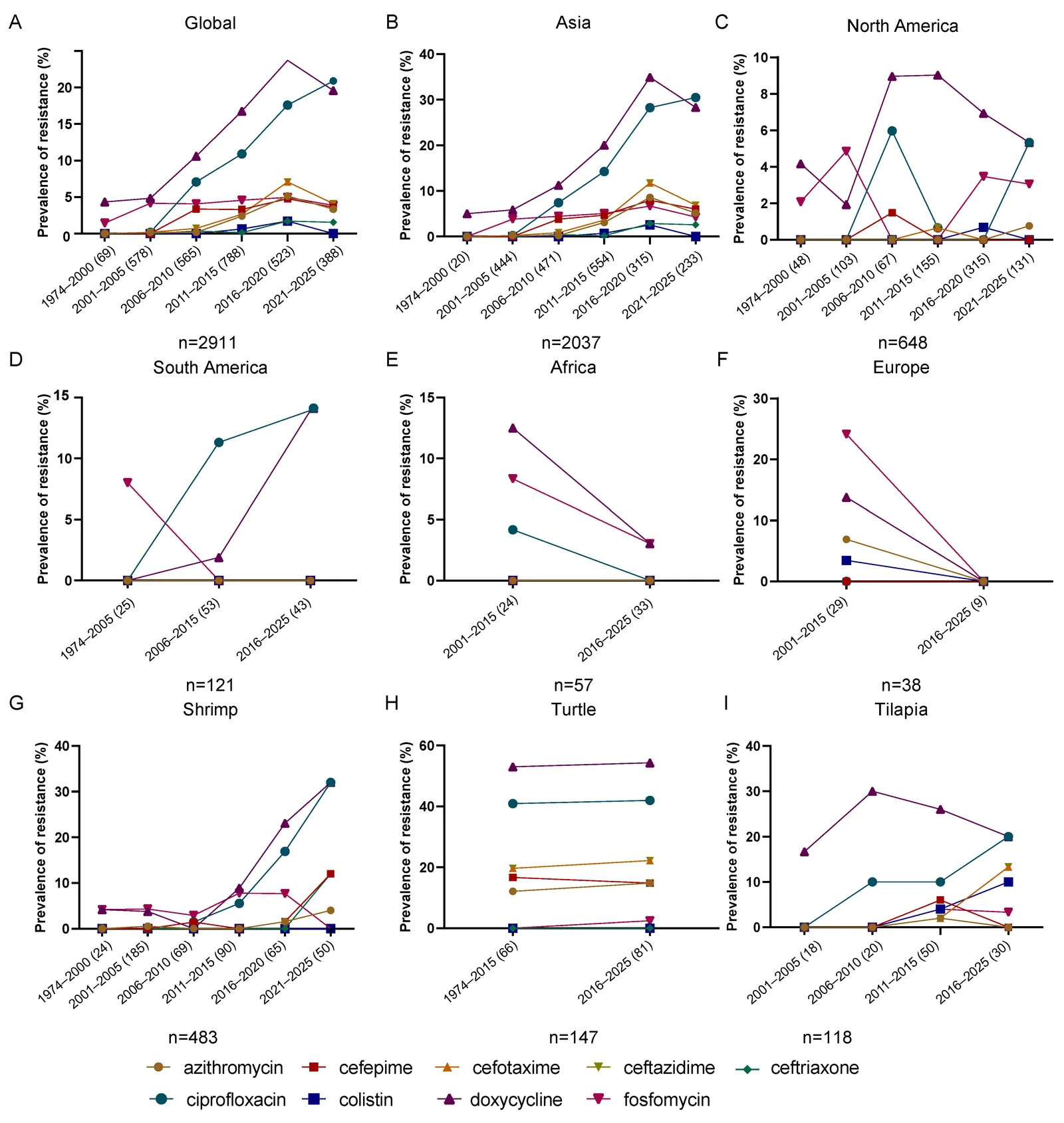
Temporal trends in clinically critical antimicrobial resistance of *Salmonella enterica* isolated from aquatic animals. (**A**) Temporal trends at the global level. (**B**) Temporal trends in Asia. (**C**) Temporal trends in North America. (**D**) Temporal trends in South America. (**E**) Temporal trends in Africa. (**F**) Temporal trends in Europe. (**G**) Temporal trends in shrimp isolates. (**H**) Temporal trends in turtle isolates. (**I**) Temporal trends in tilapia isolates. The number of isolates (n) for each time interval is labelled for each period.

Globally, overall resistance rates increased significantly over time, accompanied by an expansion in resistance phenotypes (Figure 2A). Isolates from Asia showed a resistance trend similar to the global pattern, with a slightly higher peak resistance rate (Figure 2B). *Salmonella* isolates from North America exhibited low to moderate resistance levels, with fluctuating resistance rates and no sustained increase over time. Doxycycline resistance remained relatively stable, with minor fluctuations, reaching a peak of 9.03% during 2011–2015 before declining (Figure 2C). Isolates from South America showed low overall resistance levels, while ciprofloxacin and doxycycline resistance displayed the most obvious increasing trends (Figure 2D). In contrast, resistance rates among isolates from Africa and Europe showed decreasing trends (Figure 2F). Ciprofloxacin resistance increased over time among isolates from Asia and South America but decreased among African isolates. Resistance rates to cefepime, cefotaxime, and ceftazidime increased initially and then declined among isolates from Asia and North America. No resistance to these three antibiotics was detected among isolates from South America, Africa, and Europe. Azithromycin-resistant isolates were detected in North America during 2021–2025, with a resistance rate of 0.76% (Figure 2).

AMR trends were further analyzed among *Salmonella* isolates from three major aquatic animal hosts. Isolates recovered from shrimp, turtles, and tilapia showed an overall increasing trend in resistance rates. Resistance to cefotaxime, ceftazidime, and ciprofloxacin increased among isolates from all three hosts. Doxycycline resistance increased among shrimp and turtle isolates (Figure 2G,H), whereas it increased initially and then declined among tilapia isolates, reaching the highest level during 2006–2010 (Figure 2I). Colistin resistance was detected only among tilapia isolates and showed a continuous increase during 2011–2025 (Figure 2I).

**Figure 3:**
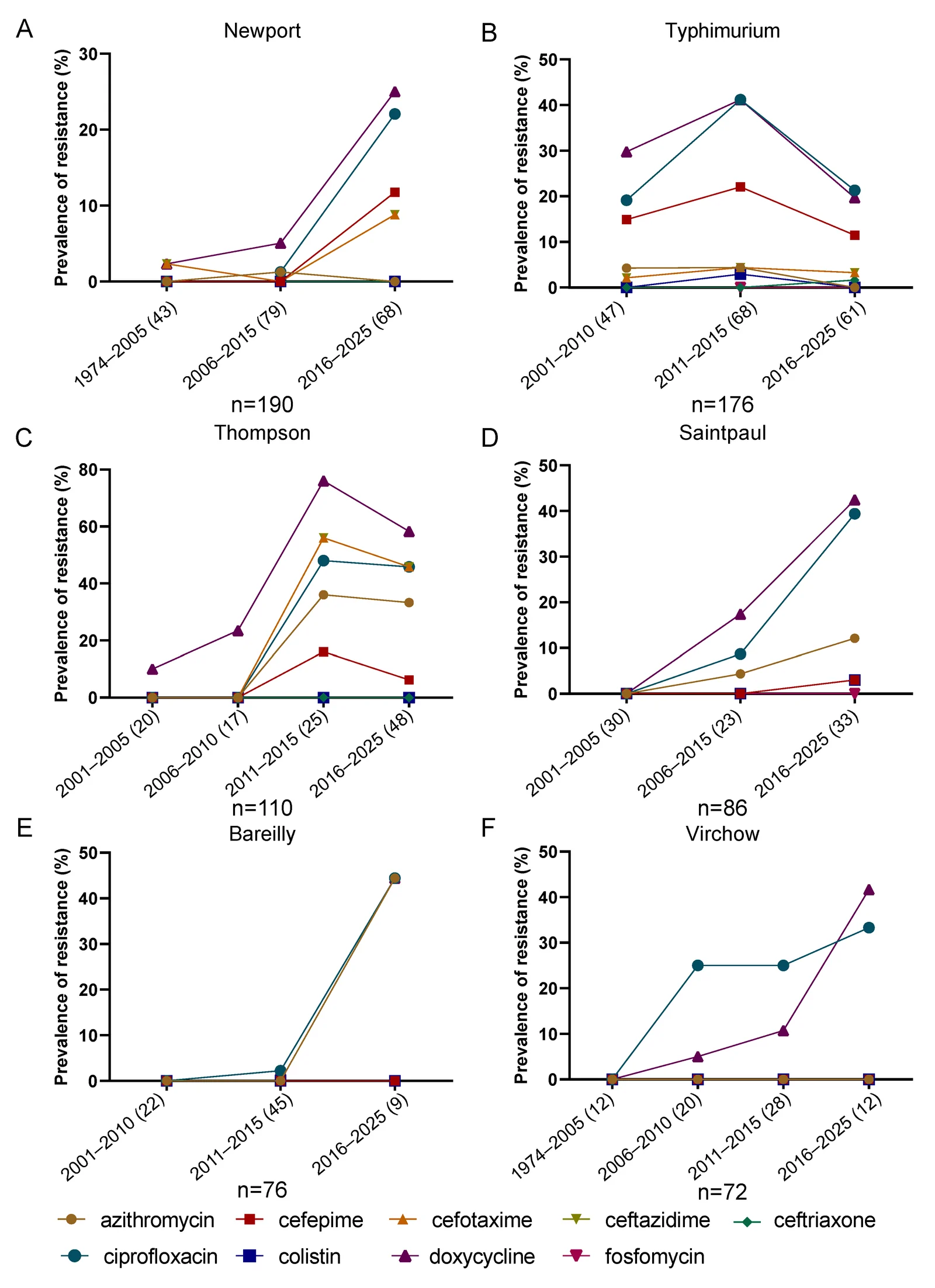
Temporal variation in clinically relevant antimicrobial resistance across the dominant *Salmonella enterica* serovars originating from aquatic animals. (**A**) Temporal trends of antimicrobial resistance in *S*. Newport. (**B**) Temporal trends of antimicrobial resistance in *S*. Typhimurium. (**C**) Temporal trends of antimicrobial resistance in *S*. Thompson. (**D**) Temporal trends of antimicrobial resistance in *S*. Saintpaul. (**E**) Temporal trends of antimicrobial resistance in *S*. Bareilly. (**F**) Temporal trends of antimicrobial resistance in *S*. Virchow. The number of isolates (n) for each time interval is labelled for each period.

AMR trends among different *Salmonella* serovars were further investigated (Figure 3). For *S*. Newport, resistance was detected only to cefotaxime, ceftazidime, and doxycycline during 1974–2005, with identical resistance rates of 2.33% for all three antibiotics. A rapid increase in resistance was observed after 2015, with resistance rates to doxycycline and ciprofloxacin reaching 25.00% and 22.06%, respectively (Figure 3A). The highest AMR levels of *S*. Typhimurium were observed during 2011–2015, when resistance rates to ciprofloxacin, doxycycline, and cefepime reached their maximum values. Resistance to most antibiotics decreased during 2016–2025, whereas ceftriaxone resistance was detected for the first time during this period (Figure 3B). The resistance profile of *S*. Thompson increased initially and then declined, with doxycycline resistance reaching the highest level of 76.00% during 2011–2015 (Figure 3C). No resistant isolates of *S*. Saintpaul were identified during 2001–2005. Low-level resistance to azithromycin, ciprofloxacin, and doxycycline was observed during 2006–2015, followed by a marked increase in resistance during 2016–2025. During this period, resistance rates to ciprofloxacin and doxycycline increased substantially, and resistance to cephalosporins and colistin was detected for the first time (Figure 3D). No resistance to the tested antibiotics was detected in *S*. Bareilly during 2001–2010. Low resistance rates to ciprofloxacin and doxycycline (2.22%) were observed during 2011–2015. From 2016 to 2025, resistance rates to azithromycin, ciprofloxacin, and doxycycline increased markedly, reaching 44.44%. No resistance to cephalosporins, colistin, or fosfomycin was detected in this serovar throughout the study period (Figure 3E). *S*. Virchow showed a slight increase in resistance during 2011–2015, with doxycycline resistance reaching 10.71% and ciprofloxacin resistance remaining at 25.00%. AMR increased substantially during 2016–2025, with doxycycline resistance rising to 41.67% and ciprofloxacin resistance reaching 33.33% (Figure 3F). In addition, no fosfomycin resistance was detected among any of the above serovars.

### 3.4. Global Trends of Clinically Important ARGs in Salmonella from Aquatic Animals

**Figure 4:**
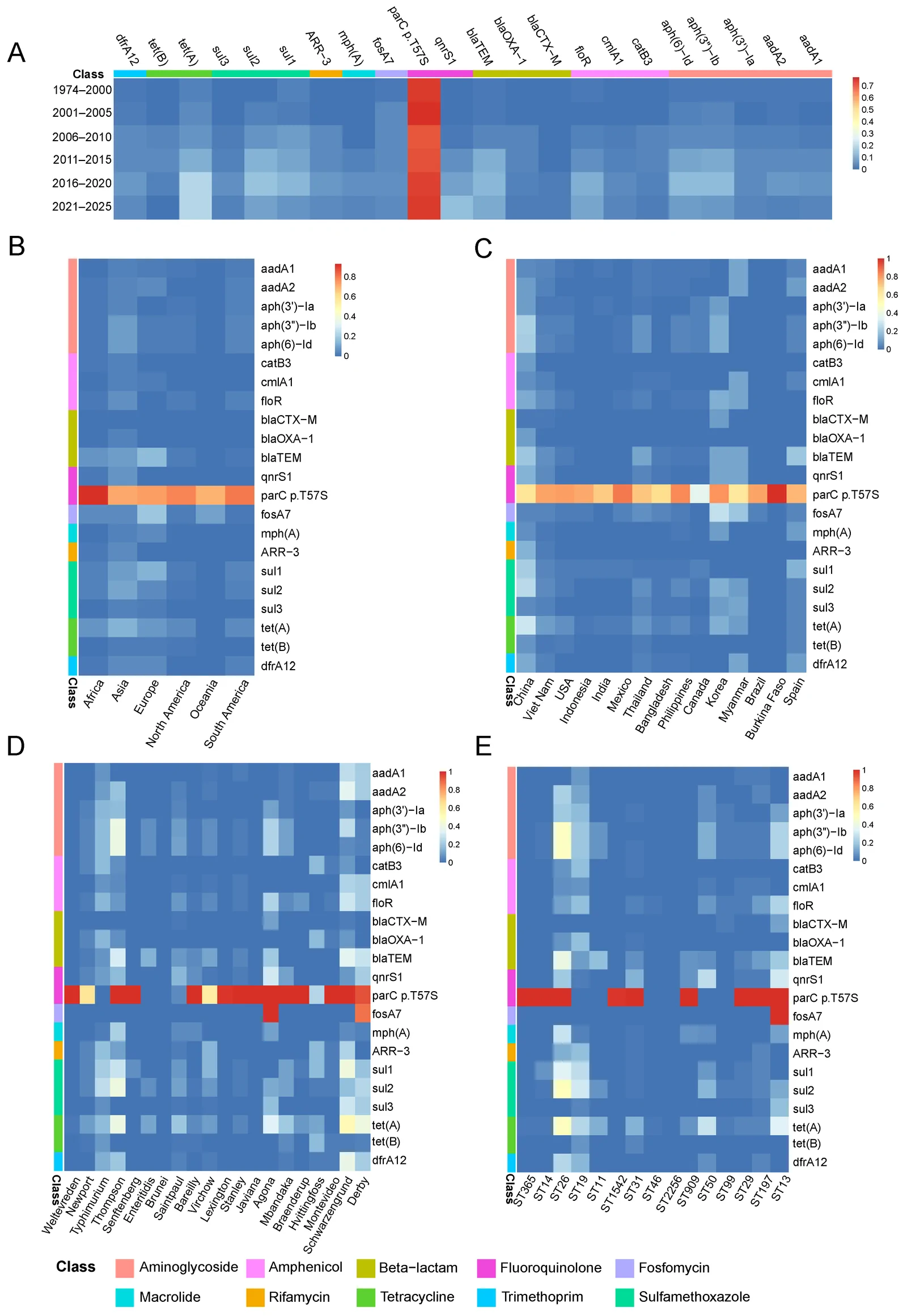
Variations in ARGs across different temporal periods, continents, countries, serovars, and STs. (**A**) Distribution of ARGs across different temporal periods. (**B**) Distribution of ARGs across different continents. (**C**) Distribution of ARGs across different countries. (**D**) Distribution of ARGs among dominant serovars. (**E**) Distribution of ARGs among dominant STs.

A total of 111 acquired ARGs and 10 *Salmonella*-specific chromosomal mutations associated with AMR were identified (Supplementary Table S4). The overall prevalence of ARGs in *Salmonella* isolates recovered from aquatic animals increased over the study period. A gradual increase in the carriage of fluoroquinolone resistance gene (*qnrS1*), aminoglycoside resistance genes (*aph(3″)-Ib*, *aph(6)-Id*), sulfonamide resistance genes (*sul1*, *sul2*), and tetracycline resistance genes (*tet*(A), *tet*(B)) was observed from 2001 to 2010 (Figure 4A).

The detected *bla*_CTX-M_ genes were mainly identified in *Salmonella* isolates recovered from aquatic animals in Asia (Figure 4B). The overall prevalence of the fosfomycin resistance gene *fosA7* was 4.26% among all isolates. The highest prevalence of *fosA7* was observed among European isolates (18.42%), followed by isolates from Oceania (10.00%), Africa (5.26%), Asia (4.71%), North America (2.31%), and South America (1.65%). The prevalence of fluoroquinolone resistance genes was relatively high among African isolates, whereas European isolates showed higher prevalence of β-lactam and fosfomycin resistance genes. Overall, the prevalence of ARGs among isolates from the Americas was lower than that among Asian isolates. Fluoroquinolone and tetracycline resistance genes were detected at moderate prevalence levels, whereas other ARG categories showed relatively low prevalence (Figure 4B). The *bla*_OXA-1_ gene was detected only in isolates from Asia and Oceania, with prevalence rates of 3.24% and 0.15%, respectively. The β-lactam resistance gene *bla*_CTX-M_ was mainly identified among Asian isolates, with a prevalence of 0.83%, and showed a low overall prevalence worldwide. Among all geographic regions, Asian isolates showed the highest prevalence of the *floR* gene (6.33%), followed by isolates from South America (3.31%), North America (2.16%), and Africa (1.75%). The *ARR-3* gene was detected only among isolates from three continents, including Asia (5.25%), Africa (1.75%), and North America (0.15%) (Figure 4B).

In Asia (Supplementary Figure S1A), the prevalence of *qnrS1* and *tet* (B) increased over time, whereas *mph(A)*, *ARR-3*, *floR, cmlA1*, and *fosA7* showed an initial increase followed by a decline. In contrast, most acquired ARGs in Africa (Supplementary Figure S1B) and Europe (Supplementary Figure S1E) exhibited decreasing trends, while *parC* p.T57S remained consistently detected across sampling periods. In South America (Supplementary Figure S1C), *dfrA12*, *tet*(A), *sul1*, *floR*, *cmlA1*, and *aph (3′)-Ia* increased over time, whereas *fosA7* declined. In North America (Supplementary Figure S1D), *mph (A)*, *floR*, and *qnrS1* increased, while *fosA7*, *sul3*, *tet*(A), *cmlA1*, and *catB3* showed fluctuating patterns. Overall, ARGs’ temporal dynamics exhibited substantial geographic heterogeneity, with several resistance determinants showing regional persistence or distinct temporal trajectories.

### 3.5. Global Distribution of Clinically Important ARGs in Salmonella from Aquatic Animals

National distribution analysis revealed a high prevalence of ARGs in *Salmonella* isolates from several Asian countries, including China, Korea, Viet Nam, Thailand, and Myanmar. In contrast, isolates from the USA, Spain, Mexico, and the Philippines showed a moderate prevalence of ARGs (Figure 4C). In these countries, the overall prevalence of ARGs was relatively low, with moderate prevalence mainly observed for fluoroquinolone, tetracycline, and sulfonamide resistance genes.

Statistical analysis characterized the distribution profiles of clinically important ARGs among different serovars (Figure 4D) and STs (Figure 4E). For example, *fosA7* was predominantly detected in *S*. Agona ST13, whereas *mph(A)* was largely confined to *S*. Thompson ST26 (Figure 4D,E). Similarly, *qnrS1* was highly prevalent in *S*. Agona ST13, *S*. Saintpaul ST50, and *S*. Thompson ST26 (Figure 4D,E). The prevalence of *aph(3″)-Ib*, *tet*(A), *sul2*, *bla*_TEM_, and *qnrS1* was 48.31%, 49.44%, 50.56%, 39.33%, and 20.22%, respectively, conferring resistance to aminoglycosides, tetracyclines, sulfonamides, β-lactams, and fluoroquinolones.

**Figure 5:**
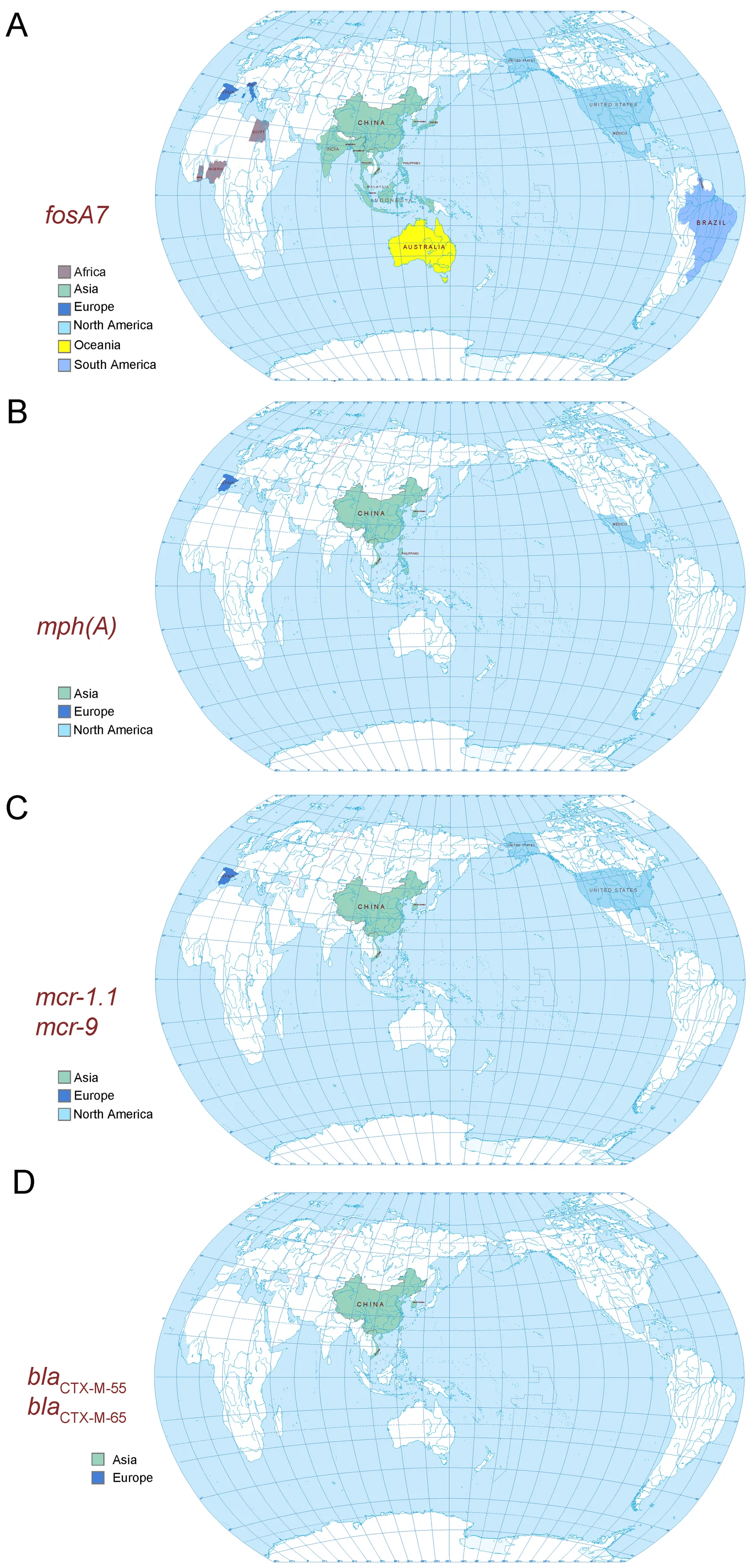
Geographical distribution of various ARGs in aquatic *Salmonella*. Countries where each target gene was not detected are not color-coded on the map. (**A**) Geographical distribution of the fosfomycin resistance gene, *fosA7*. (**B**) Geographical distribution of the azithromycin resistance gene, *mph(A).* (**C**) Geographical distribution of mobile colistin resistance genes, *mcr-1.1* and *mcr-9*. (**D**) Geographical distribution of β-lactamase resistance genes, *bla*_CTX-M-55_ and *bla*_CTX-M-65_. The base map is adapted from the standard map of the Ministry of Natural Resources of China (Map review No. GS (2021) 5449), with national boundary features unmodified.

In addition, the global distribution of clinically important ARGs in *Salmonella* isolates from aquatic animals was analyzed. The fosfomycin resistance gene *fosA7* was detected in isolates from 22 countries, with the highest prevalence observed in Korea (27.27%), followed by Myanmar (19.05%) and Thailand (6.09%) (Figure 5A). The azithromycin resistance gene *mph(A)* was identified in isolates from six countries, with the highest prevalence in Spain (10.00%), followed by Korea (9.09%), China (6.79%), Viet Nam (1.72%), the Philippines (1.49%), and Mexico (0.74%) (Figure 5B). The mobile colistin resistance genes *mcr-9* and *mcr-1.1* were detected in isolates from five countries, including Korea (9.09%), Spain (5.00%), China (0.46%), the USA (0.24%), and Viet Nam (0.22%) (Figure 5C). The β-lactam resistance genes *bla*_CTX-M-55_ and *bla*_CTX-M-65_ were identified in isolates from three countries, with the highest prevalence observed in Korea (9.09%), followed by China (1.23%) and Viet Nam (1.08%) (Figure 5D).

## 4. Discussion

In this study, we established a global genomic dataset of 2911 aquatic-derived *Salmonella enterica* isolates collected between 1974 and 2025 from 67 countries across six continents, representing 194 serovars and 364 STs. However, genomic investigations of aquatic-derived *Salmonella* remain limited compared with those of human- and terrestrial animal-associated populations, with previous studies largely focusing on specific countries, individual aquatic products, or relatively small isolate collections [20,43]. In addition, several studies have characterized AMR profiles and ARGs in aquatic isolates [5,20,30]; resistance to WHO-recommended priority antibiotics in aquatic-derived *Salmonella* remains comparatively underexplored, in sharp contrast to the well-documented genomic epidemiology of clinical invasive *Salmonella* from human bloodstream infections [44,45].

In this work, we observed *S*. Weltevreden, *S*. Newport, and *S*. Typhimurium as the three dominant serovars, and ST365, ST14, ST26, ST19, and ST11 were the five most prevalent STs. A previous survey of aquatic food-derived *Salmonella* in China identified 88 serovars and 100 STs, with *S*. Thompson ST26, *S*. Newport ST46, and *S*. Derby ST40 as the predominant lineages [20]. In contrast, our dataset covers a much broader geographic range and captures substantially greater serovar and ST diversity, providing a more comprehensive resource for investigating the global epidemiology and AMR of aquatic-derived *Salmonella.* Our dataset provides a valuable genomic resource for understanding the worldwide distribution and long-term evolution of clinically important AMR and supports future genomic surveillance within the One Health framework. To our knowledge, this study represents the largest-scale global genome-based assessment focusing on resistance to the WHO’s medically important antimicrobials among aquatic-derived *Salmonella*.

In recent years, the rapid expansion of global aquaculture has resulted in increased antimicrobial use for disease prevention and treatment in aquatic animals [46]. This practice may create selective pressures that facilitate the emergence and dissemination of AMR among aquatic-derived *Salmonella*. A one-year surveillance study of *Salmonella* in shrimp from retail markets across China reported an overall isolation rate of 10.10% [47]. Numerous isolates exhibited MDR and were frequently detected in both freshwater and marine aquatic products, highlighting the potential risk of AMR transmission through aquatic food products [20]. In the present study, the increasing trend of ciprofloxacin resistance coincided with the widespread distribution of the *parC* p.T57S point mutation, which was detected at a high prevalence (70–77%) among aquatic-derived *Salmonella* isolates. Therefore, region-specific and serovar-focused AMR surveillance systems are needed to improve risk assessment and support targeted antimicrobial management.

Aquatic-derived *Salmonella* isolates in our dataset showed an overall increasing trend in AMR over time. Similar increasing trends in AMR have been reported in aquaculture systems and food-producing animals, likely reflecting the cumulative effects of antimicrobial exposure and selection pressure associated with intensive production practices [24,28]. Isolates showed uneven temporal distribution, with merely 2.37% collected during 1974–2000 and peak sampling (27.07%) occurring in 2011–2015. This pattern reflects cumulative deposition of public *Salmonella* genomes rather than genuine epidemiological shifts in pathogen prevalence. Despite reductions in resistance to individual antimicrobial agents following antimicrobial stewardship and regulatory interventions, MDR populations may persist through the expansion of successful clones, environmental reservoirs, and MGEs [12,18,48]. We adopted a merged early time window followed by sequential five-year strata to maintain analytical robustness. Temporal fluctuations in continental ARG prevalence coincided with landmark global AMR policies, including the 2006 EU ban on antimicrobial growth promoters [49] and the 2017 WHO antimicrobial stewardship guidelines [50]. Nevertheless, direct causal links cannot be inferred between these policy measures and observed resistance trends, due to uneven temporal sampling, heterogeneous national policy enforcement, and clonal expansion and dissemination of dominant *Salmonella* lineages. In our dataset, isolates collected between 1974 and 2000 exhibited relatively low resistance levels, whereas ARG carriage increased gradually during 2001–2020. This temporal pattern is consistent with previous observations of increasing AMR trends in aquaculture systems, particularly in Asia, where intensive aquaculture production is common [28]. Therefore, continuous genomic surveillance and long-term antimicrobial management remain essential for controlling the persistence and dissemination of MDR *Salmonella*.

Compared with a previous global surveillance study [27], our study further revealed regional differences in AMR patterns among aquatic-derived *Salmonella*. These regional patterns should nevertheless be interpreted cautiously because country-specific resistance estimates may be affected by differences in sampling intensity and population structure. The relatively high resistance rates observed in some countries, including China and Korea, may partly reflect uneven sampling and the overrepresentation of specific lineages or sampling events in public databases. Although several resistant lineages and STs were identified in these regions, the current dataset does not contain sufficient epidemiological information to determine whether these patterns resulted from local clonal expansion, outbreak-associated transmission, or broader dissemination of resistance determinants. The previous study analyzed resistance patterns of *Salmonella* and *Escherichia coli* from food animals using geospatial models and identified Asia, Africa, and South America as regions with relatively high AMR risk [27]. The resistance patterns observed in our study were generally consistent with these predictions. However, isolates from Africa showed relatively low overall resistance levels in our dataset, which differed from the previously predicted regional hotspots [27]. This discrepancy may be explained by the limited number of aquatic-derived *Salmonella* isolates available from Africa, which accounted for only 1.96% of the total dataset. In addition, differences in sampling strategies and production systems may also contribute to the observed regional variation. Therefore, expanded surveillance and targeted sampling of aquatic environments in Africa are needed to better characterize global AMR distributions.

At the serovar level, *S*. Weltevreden was the predominant serovar among aquatic-derived *Salmonella* worldwide, accounting for 15.53% of all isolates. However, its AMR burden was not the highest among the analyzed serovars. In contrast, *S*. Thompson displayed a pronounced MDR phenotype, consistent with recent genomic investigations describing the emergence and dissemination of clinically important MDR *S*. Thompson ST26 lineages [26]. In the present study, *S*. Thompson exhibited 100% resistance to several clinically important antimicrobials, which was substantially higher than previously reported levels [26]. A recent genomic study analyzed 141 *S*. Thompson ST26 isolates collected from clinical samples and animal-derived foods across China between 1997 and 2020, reporting resistance rates of 72.3% for tetracycline, 63.1% for cefotaxime, and 24.8% for ciprofloxacin [26]. The higher resistance levels observed in our dataset may reflect the continued circulation of highly resistant *S*. Thompson ST26 lineages after 2020, suggesting the persistence of this high-risk lineage in recent years. Resistance determinants, including *mph(A)*, *qnrS1*, and 3GCs’ resistance genes, have been reported in MDR *S*. Thompson ST26 isolates and may contribute to their resistance profiles [26,51]. Previous studies have identified IncC plasmids carrying multiple resistance determinants among *S*. Thompson isolates recovered from seafood and human diarrheal patients [51]. However, our research was designed to focus on the epidemiological characteristics of AMR, so we did not explore the presence and genetic context of such plasmids in this study. More broadly, previous genomic studies have suggested that MGEs, particularly plasmids, play important roles in shaping the *Salmonella* resistome by facilitating the acquisition and spread of AMR determinants across diverse lineages and ecological reservoirs [7,25,31,32,52]. These findings underscore the importance of aquatic-associated reservoirs as potential niches for the maintenance and dissemination of high-risk MDR *Salmonella* lineages.

This study has several limitations that should be acknowledged. First, regional and temporal AMR prevalence estimates may be biased owing to uneven, non-random availability of public *Salmonella* genomes. These datasets were generated by independent research groups adopting inconsistent sampling frameworks rather than unified protocols, which exacerbates sampling bias. Approximately 70% of strains originated from Asia, whereas isolates from Africa, Europe, and Oceania remain scarce, potentially compromising AMR prevalence estimates for these under-sampled regions. In addition, temporal shifts in the composition of serovars, STs, and sample sources may distort observed AMR trends. Although stratified analyses based on major serovars and STs were performed, residual sampling bias cannot be fully excluded. Second, AMR phenotypes in this study were predicted based on genomic data rather than determined by antimicrobial susceptibility tests. Although ResFinder and PointFinder are widely utilised in WGS-based *Salmonella* surveillance, genotype–phenotype discordance may arise from incomplete genotype–resistance associations, variable gene expression, regulatory mechanisms or newly emerging resistance pathways. Such mismatches are particularly relevant to low-prevalence resistance traits and chromosomal mutations; therefore, our AMR profiles should be interpreted as genomic predictions rather than experimentally validated phenotypes. Third, this study could not fully distinguish the relative contributions of selective pressures from aquaculture, clinical settings, and environmental sources. In particular, a lack of aquaculture antimicrobial consumption data precludes direct evaluation of associations between antimicrobial exposure and observed resistance patterns. Furthermore, the influences of non-antimicrobial factors on resistance evolution among aquatic-associated *Salmonella* remain poorly characterised. Fourth, this study mainly focused on the epidemiological distribution of acquired ARGs. All genomic data relied on short-read sequencing, and fragmented assemblies hinder reliable reconstruction of plasmid backbones and the intact genetic contexts of resistance genes. Plasmid typing and MGE association analyses of key ARGs were therefore not conducted, which limits further mechanistic interpretation of ARG dissemination. Future surveillance efforts should expand global sampling across Africa, Europe and Oceania to construct geographically balanced, more representative datasets for cross-continental and global AMR epidemiological research. Longitudinal investigations integrating phenotypic AST validation, multifactorial environmental metadata, and long-read sequencing will help resolve the genetic environments of critical resistance genes and further elucidate the drivers governing AMR emergence and spread within aquatic ecosystems.

## 5. Conclusions

This study uncovers the global spatiotemporal evolution of population structure and WHO-recommended AMR in aquatic-derived *Salmonella* using a large-scale genomic dataset collected between 1974 and 2025. To the best of our knowledge, this is the first-ever comprehensive analysis leveraging the largest genomic dataset worldwide to elucidate priority AMR in aquatic animals. Global clinically important AMR levels, which showed an overall increasing trend, with isolates from Asia, particularly China, showing a higher prevalence of resistance to several important antibiotics. AMR profiles varied substantially among different serovars and STs, with distinct resistance patterns observed among major lineages. It should be noted that these findings require cautious interpretation. Although we have analysed all accessible public genomic data, regional and temporal estimates may be biased owing to uneven, non-random sampling of *Salmonella* genomes. Furthermore, AMR phenotypes were predicted genomically without phenotypic susceptibility validation. Integrated surveillance involving aquaculture, environmental monitoring, and clinical settings is needed to better understand AMR evolution in aquatic animals.

## Figures and Tables

**Table 1:** Differences in priority antimicrobial resistance of aquatic animal *Salmonella* by geographical location. Data are presented as resistance rates (%); cells with the highest resistance rate in each column are shaded to improve readability.

	Macrolide	4GCs	3GCs	3GCs	3GCs	Fluoroquinolone	Colistin	Tetracycline	Fosfomycin
Category (Count)	Azithromycin	Cefepime	Cefotaxime	Ceftazidime	Ceftriaxone	Ciprofloxacin	Colistin	Doxycycline	Fosfomycin
Total (2911)	2.10	2.89	2.71	2.71	0.62	10.31	0.48	14.53	4.29
Africa (57)	0	0	0	0	0	1.75	0	7.02	5.26
Asia (2037)	2.85	4.07	3.83	3.83	0.88	13.50	0.59	18.02	4.76
Europe (38)	5.26	0	0	0	0	0	2.63	10.53	18.42
North America (648)	0.15	0.15	0.15	0.15	0	1.85	0.15	6.33	2.31
Oceania (10)	0	0	0	0	0	0	0	0	10.00
South America (121)	0	0	0	0	0	9.92	0	5.79	1.65
China (648)	6.79	10.96	10.03	10.03	1.23	32.10	0.93	41.36	4.17
Viet Nam (464)	1.94	1.51	1.08	1.08	1.08	9.91	0.43	11.21	4.31
USA (413)	0	0.24	0.24	0.24	0	1.69	0.24	8.47	2.42
Indonesia (275)	0	0.36	0.36	0.36	0.36	0	0	1.09	2.55
India (222)	0	0	0.90	0.90	0	0.45	0	2.70	0.90
Mexico (136)	0.74	0	0	0	0	3.68	0	2.94	3.68
Thailand (115)	0	0	0	0	0	6.96	0	12.17	6.09
Bangladesh (93)	0	0	0	0	0	1.08	0	3.23	4.30
Philippines (67)	1.49	0	0	0	0	0	0	5.97	2.99
Canada (55)	0	0	0	0	0	0	0	3.64	0
Korea (44)	9.09	9.09	11.36	11.36	9.09	11.36	9.09	13.64	27.27
Myanmar (42)	0	0	0	0	0	11.90	0	9.52	19.05
Brazil (28)	0	0	0	0	0	10.71	0	0	3.57
BurkinaFaso (22)	0	0	0	0	0	0	0	0	0
Spain (20)	10.00	0	0	0	0	0	5.00	10.00	5.00

**Table 2:** Differences in priority antimicrobial resistance of aquatic animal *Salmonella* by serovars and STs. Data are presented as resistance rates (%); cells with the highest resistance rate in each column are shaded to improve readability.

	Macrolide	4GCs	3GCs	3GCs	3GCs	Fluoroquinolone	Colistin	Tetracycline
Category (Count)	Azithromycin	Cefepime	Cefotaxime	Ceftazidime	Ceftriaxone	Ciprofloxacin	Colistin	Doxycycline
Weltevreden (452)	0	0	0	0	0	0	0	1.11
Newport (190)	0.53	4.21	3.68	3.68	0	8.42	0	11.58
Typhimurium (176)	2.84	16.48	3.41	3.41	0.57	28.41	1.14	30.68
Thompson (110)	22.73	6.36	32.73	32.73	0	30.91	0	48.18
Senftenberg (107)	0	0	0	0	0	2.80	0	0.93
Enteritidis (90)	0	1.11	1.11	1.11	1.11	0	0	11.11
ST365 (447)	0	0	0	0	0	0	0	1.12
ST14 (97)	0	0	0	0	0	3.09	0	1.03
ST26 (89)	28.09	7.87	40.45	40.45	0	38.20	0	59.55
ST19 (84)	4.76	17.86	3.57	3.57	0	25.00	0	40.48
ST11 (76)	0	1.32	1.32	1.32	1.32	0	0	13.16

## Data Availability

The data presented in this study are available in [NCBI] at [https://www.ncbi.nlm.nih.gov/]. These data were derived from the following resources available in the public domain: https://www.ncbi.nlm.nih.gov/.

## Supplementary Materials

The following supporting information can be downloaded at: https://www.mdpi.com/article/10.3390/vetsci13090868/s1, Figure S1. Temporal variations in ARGs at the continental scale. Major global antimicrobial policy milestones: 2006 (EU ban on antimicrobial growth promoters), 2017 (WHO guidelines for antimicrobial use in food-producing animals). (A) Temporal distribution of ARGs in Asia. (B) Temporal distribution of ARGs in Africa. (C) Temporal distribution of ARGs in South America. (D) Temporal distribution of ARGs in North America. (E) Temporal distribution of ARGs in Europe; Table S1. Metadata and Accession Numbers of *Salmonella* Isolates; Table S2. Summary statistics of genome assemblies generated by QUAST v5.3.0; Table S3. Antibiotic resistance genes and gene mutations detected in 2911 *Salmonella* isolates; Supplementary Table S4. Frequency of antibiotic resistance genes; All datasets for Table S1–S3 are provided in the single uploaded Excel file Supplementary_Tables_S1-S3.xlsx.

## Abbreviations

The following abbreviations are used in this manuscript:

AMR	antimicrobial resistance
STs	sequence types
MDR	multidrug resistant
ARGs	antibiotic resistance genes
MLST	Multilocus Sequence Typing
3GCs	third-generation cephalosporins
MGEs	mobile genetic elements
