# Supplementary material for: Global Genomic Analysis Reveals Spatial Patterns and Temporal Trends of Priority-Antimicrobial Resistance in Salmonella enterica Isolates from Aquatic Animals
Source: Vet Sci. 2026 Aug 26;13(9):868. doi: 10.3390/vetsci13090868 (PMC13611974; doi:10.3390/vetsci13090868)
Supplement: Supplementary file 1 [file vetsci-13-00868-s001.zip › 20260811-Supplementary information.pdf]

Global genomic analysis reveals spatial patterns and temporal trends of priority-antimicrobial resistance in *Salmonella* isolates from aquatic animals

Ziru Yang[1,2,#], Shuqi Yan[1,#], Limin Zhang[1], Chaochao Zhao[1], Jiangtao Wang[1], Jiale Chen[1], Yue Liu[3], Xuebin Xu[3,*], Yanan Wang[1,2,4,*]

[1] International Joint Research Center of National Animal Immunology, College of Veterinary Medicine, Henan Agricultural University, Zhengzhou, Henan, China.

[2] Longhu Laboratory of Advanced Immunology, Zhengzhou, Henan, China.

[3] Division of Pathogen Testing and Analysis, Shanghai Municipal Center for Disease Control and Prevention, Shanghai, China.

[4] Beijing Key Laboratory of Antimicrobial-Resistant Pathogen Microbiology and AI-Empowered Containment, Institute of Microbiology, Chinese Academy of Sciences (CAS), Beijing, China.

[#]These authors contributed equally to this work.

*E-mail: wangyanan1001@henau.edu.cn (YNW), xuxuebin@scdc.sh.cn (XBX)

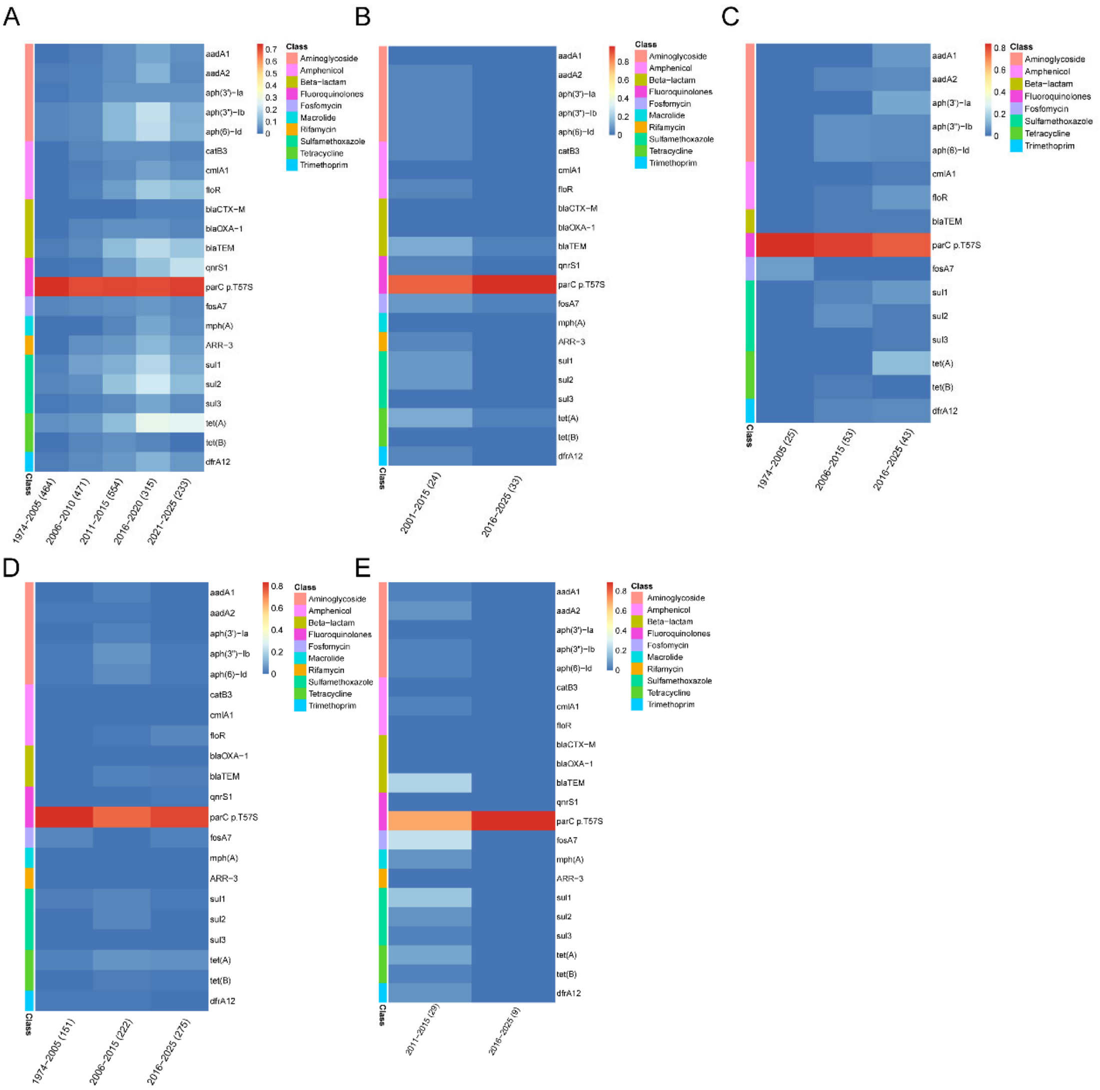

Supplementary Figure S1. Temporal variations of ARGs at the continental scale. Major global antimicrobial policy milestones: 2006 (EU ban on antimicrobial growth promoters), 2017 (WHO guidelines for antimicrobial use in food-producing animals). (A) Temporal distribution of ARGs in Asia. (B) Temporal distribution of ARGs in Africa. (C) Temporal distribution of ARGs in South America. (D) Temporal distribution of ARGs in North America. (E) Temporal distribution of ARGs in Europe.
